# Metabolomic Profiling of Drug Responses in Acute Myeloid Leukaemia Cell Lines

**DOI:** 10.1371/journal.pone.0004251

**Published:** 2009-01-22

**Authors:** Stefano Tiziani, Alessia Lodi, Farhat L. Khanim, Mark R. Viant, Christopher M. Bunce, Ulrich L. Günther

**Affiliations:** 1 CR UK Institute for Cancer Studies, University of Birmingham, Henry Wellcome Building for Biomolecular NMR Spectroscopy (HWB-NMR), Birmingham, United Kingdom; 2 School of Biosciences, University of Birmingham, Birmingham, United Kingdom; Dresden University of Technology, Germany

## Abstract

Combined bezafibrate (BEZ) and medroxyprogesterone acetate (MPA) exert unexpected antileukaemic activities against acute myeloid leukaemia (AML) and these activities are associated with the generation of reactive oxygen species (ROS) within the tumor cells. Although the generation of ROS by these drugs is supported by preceding studies including our own, the interrelationship between the cellular effects of the drugs and ROS generation is not well understood. Here we report the use of NMR metabolomic profiling to further study the effect of BEZ and MPA on three AML cell lines and to shed light on the underlying mechanism of action. For this we focused on drug effects induced during the initial 24 hours of treatment prior to the onset of overt cellular responses and examined these in the context of basal differences in metabolic profiles between the cell lines. Despite their ultimately profound cellular effects, the early changes in metabolic profiles engendered by these drugs were less pronounced than the constitutive metabolic differences between cell types. Nonetheless, drug treatments engendered common metabolic changes, most markedly in the response to the combination of BEZ and MPA. These responses included changes to TCA cycle intermediates consistent with recently identified chemical actions of ROS. Notable amongst these was the conversion of α-ketoglutarate to succinate which was recapitulated by the treatment of cell extracts with exogenous hydrogen peroxide. These findings indicate that the actions of combined BEZ and MPA against AML cells are indeed mediated downstream of the generation of ROS rather than some hitherto unsuspected mechanism. Moreover, our findings demonstrate that metabolite profiles represent highly sensitive markers for genomic differences between cells and their responses to external stimuli. This opens new perspectives to use metabolic profiling as a tool to study the rational redeployment of drugs in new disease settings.

## Introduction

Drug discovery is slow and prohibitively expensive resulting in low rates of novel agents reaching the clinic [1]–[3]. The cost of developing new drugs naturally deters the development of drugs in less common diseases where profitability will be lower and excludes poorer nations from benefiting from those drugs that do come to market. One approach to alleviating this problem is to develop a better understanding of old drugs that will permit their rational redeployment in new disease settings [1]. Metabolite concentrations represent sensitive markers of genomic changes and responses of cells and tissues to external stimuli [4]–[9]. Consequently, the development of robust metabolomic platforms will greatly facilitate the understanding of the *in vitro* and *in vivo* actions of available drugs and aid their incorporation into novel therapeutic settings [10]–[13]. ^1^H-NMR spectroscopy permits highly reproducible chemical analyses that can often be correlated to *in vivo* NMR spectra. Here we have analysed the metabolic response profiles of acute myeloid leukemia (AML) cell lines towards drugs not conventionally considered as leukemia agents.

The AMLs are a genetically heterogeneous group of cancers characterized by the abnormal maturation, survival and proliferation of myeloid cells in bone marrow [14]. The concurrent loss of normal hemapoiesis results in life threatening insufficiency of normal white and red blood cells, and platelets. Consequently if untreated, patients suffer from anemia and increased risk of infection and bleeding, and often die within weeks of diagnosis. Although improved chemotherapy has benefited younger patients the majority of patients still succumb to their disease [15]. What is required are therapies that exert an antileukemic affect without being myeloablative or systemically toxic.

We and others have previously shown the anti-leukemic actions of the lipid lowering drug Bezafibrate (BEZ) and the contraceptive steroid medroxyprogesterone acetate (MPA) against AML cells [16], [17] and have shown the more potent action of the drugs when combined [18], [19]. Treatment of AML cells with combined BEZ and MPA resulted in growth cessation, and induction of differentiation and apoptosis. The improved anti-leukemic actions of combined BEZ and MPA appeared to converge on the accumulation of prostaglandin D_2_ (PGD_2_); BEZ increasing PGD_2_ synthesis and MPA inhibited its conversion to 11β-PGF_2α_ by the aldoketo reductase AKR1C3. Elevated PGD_2_ in turn gave rise to elevation of the alternative downstream and potently anti-neoplastic cyclopentanone prostaglandin 15deoxyΔ^12,14^PGJ_2_ and the consequent sustained generation of reactive oxygen species (ROS) [19]. However, the importance of ROS generation in mediating the cellular responses to combined MPA and BEZ or some other hitherto unrecognized effects of the drugs remained uncertain. In this study we employed ^1^H-NMR metabolomics to functionally assign drug induced changes in the metabolism of AML cell lines. We chose cell lines that either apoptose in response to the drugs (KG1a and K562) or that enter into differentiation (HL-60). This study identifies unsuspected effects of MPA and BEZ on metabolites including those of the TCA cycle that support the importance of ROS in mediating the drugs' effects and also highlights differences associated with either differentiation or apoptotic responses. Moreover, our study demonstrates the applicability of ^1^H-NMR metabolomics as an analytical tool for the understanding of drug-drug interactions.

## Results

### ^1^H-NMR profiles reveal distinct differences between AML cell lines

**Figure 1:**
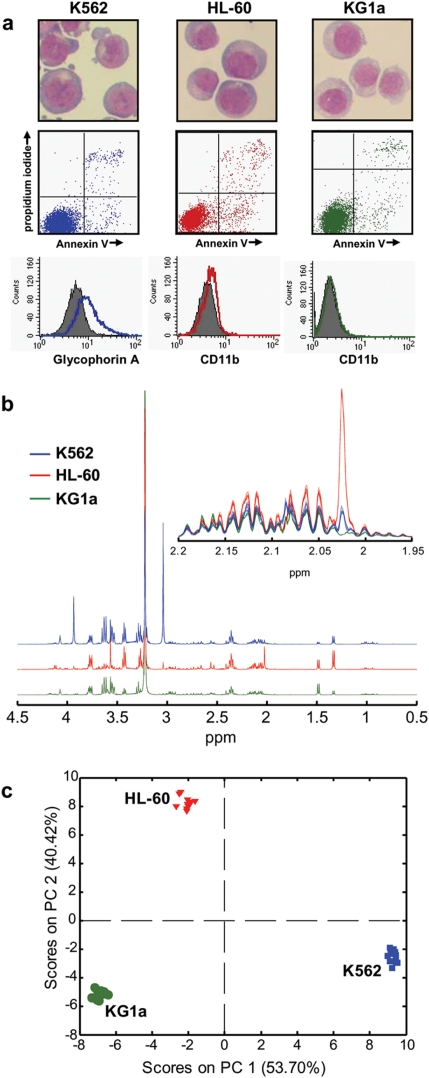
Phenotypic and metabolic differences among AML cell lines (K562, HL-60 and KG1a). (a) Cell morphology (Jenner-Giemsa stained cytospins) and fluorescence flow cytometry analysis for apoptosis (Annexin V and propidium iodide double staining) and differentiation (expression of the erythroid antigen Glycophorin A in K562 and the myeloid differentiation antigen CD11b in HL-60 and KG1a cells; sample results are compared to an isotype, grey-shaded); typical features of healthy K562, KG1a and HL-60 cells are observed (b) ^1^H-NMR spectra (expanded between 0.5–4.5 ppm and between 1.95–2.2 ppm in the small insert) collected on AML extracts of HL-60, KG1a and K562 cells (12 replicates per cell line) and (c) scores plot (PC1 vs PC2) obtained from the PCA of the NMR spectra of 12 datasets per cell line.

We first recorded ^1^H-NMR spectra of exponentially growing HL-60, KG1a and K562 cells. These cell lines represent highly undifferentiated AMLs (KG1a), more differentiated AMLs (HL-60) and AML blast crisis arising from a predisposing chronic myeloid leukaemia (CML; K562). Figure 1a shows the morphology of exponentially growing cells from these cell lines and shows that the basal rates of apoptosis and differentiation in these cell lines are low. Projected *J*-resolved NMR spectra (*J*-RES) revealed significant differences between the cell lines (Figure 1b) that were stable and highly reproducible. An expanded section (1.95–2.2 ppm) of the ^1^H-NMR spectra of the 12 replicates of each cell line for KG1a, K562 and HL-60 highlights the excellent reproducibility of the independently grown cell samples.

Multivariate statistical modeling using principal component analysis (PCA) was performed on the ^1^H-NMR spectra of 12 independently grown replicates from each of the cell lines and the scores plot (principal components 1 vs 2) resulting from PCA (Figure 1c) revealed very clear discrimination between the intracellular metabolome of the three cell lines. It must be emphasized that these differences were identified using an unsupervised analysis, *i.e.* without any prior information about the samples. Since all three cell lines were grown under identical conditions the observed discrimination demonstrates that the genetic differences between the cell lines are strongly represented by their individual metabolic profiles.

Loadings plots for the first principal component of the pairwise comparison between the three cell lines identified lactate, alanine, N-acetyl-aspartate, creatine, phosphocholine and myo-inositol as major discriminators between the cell lines (Figure S1). Lactate and N-acetyl-aspartate were both elevated in HL-60; alanine was high and creatine low in HL-60 and KG1a; phosphocholine was lower in K562 and myo-inositol had lower levels in HL-60. The ability to distinguish cell lines so accurately represents significant progress for metabolic phenotyping of cancer cells and forms a basis for profiling differing tumor backgrounds as well as shared and non shared drug responses.

### ^1^H-NMR profiles depict the response of AML cell lines to bezafibrate and medroxyprogesterone acetate

**Figure 2:**
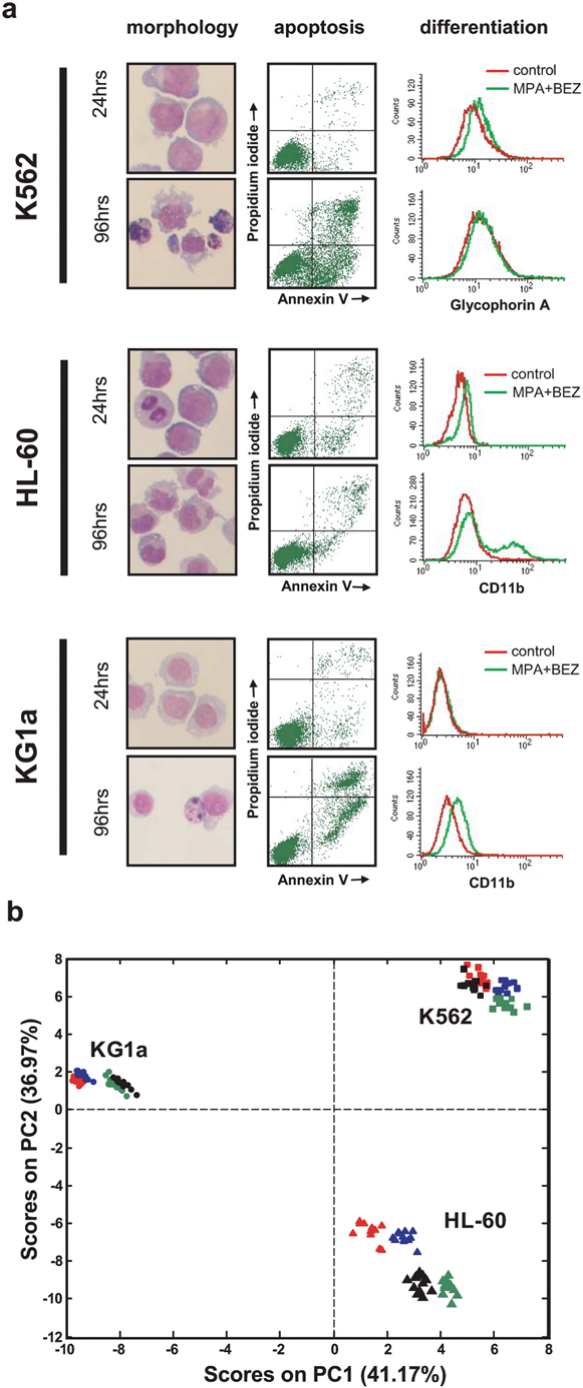
Effect of treatment with MPA, BEZ and the combination of both agents on AML cell lines (K562, HL60 and KG1a). (a) Cell morphology and flow cytometry analysis for apoptosis and differentiation are shown in K562, HL-60 and KG1a cells after 24 hrs and 96 hrs of treatment with the combination of MPA and BEZ. In these AML cell lines, neither apoptosis nor differentiation signatures are observed after a 24 hrs treatment with MPA and BEZ in combination. Prominent features of apoptosis and differentiation develop after 96 hours of treatment. (b) PCA scores plot obtained from the analysis of the NMR spectra of all the AML cell lines after 24 hrs of treatment. Solvent control (red), MPA (blue), BEZ (black), and MPA+BEZ (green) treatments are included, with 12 replicates per treatment per cell line.

Our previous studies identified antileukaemic actions of combined MPA and BEZ (MPA+BEZ) against AML cells and cell lines that result in either cessation of cell proliferation accompanied by differentiation or by entering into apoptosis [19]. In this study we wished to investigate whether a metabolomic approach could identify signatures associated with these events and whether these signatures provide new insight into the mechanistic actions of this new experimental approach. HL-60 cells were chosen as a line that responds to MPA+BEZ by entering into differentiation and KG1a and K562 as cell lines whose primary response is apoptosis. We wished to identify signatures indicative of drug responses at times prior to any marked cellular changes. Figure 2a shows that very little differentiation of HL-60 cells or apoptosis of either KG1a or K562 cells were observed after 24 hours exposure to MPA+BEZ. However these processes were subsequently evident at 96 hrs. We therefore performed our metabolomic analyses at 24 hours post treatment.

The PCA scores plot shown in Figure 2b demonstrates that exposure of the cells to the drugs alone or in combination caused changes in the metabolome of all three cell lines. It is notable that despite the ultimately profound anti-leukemic effects of the drugs the changes induced at 24 hours were less marked than the pre-existing differences determined by the genetic backgrounds of the cell lines. Alterations provoked by the drugs were greatest in HL-60 cells but remained small when compared to the inherent differences between cell types. Importantly, even in the presence of drugs, reproducibility between replicates (12 per treatment for each treatment and cell line) remained very high and sample to sample variation within each group was significantly smaller than any variation arising from treatment or genetic variability. The relatively subtle but distinct metabolic changes seen in response to drug treatments allied with the lack of overt cellular changes at 24 hours, strongly imply that we have measured genuine drug responses and not secondary changes within the metabolome occurring after drug induced changes in cell behavior.

### ^1^H-NMR profiles reveal combinatorial effects of MPA+BEZ treatment

**Figure 3:**
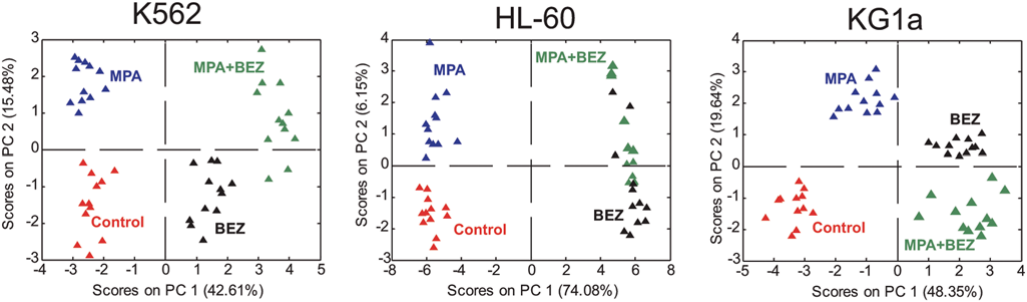
Principal component analysis (PCA) scores plots (PC1 vs PC2) including the solvent control (red symbols) and the 3 drug treatments (MPA: blue, BEZ: black, and MPA+BEZ: green symbols) for K562, HL-60, and KG1a cell lines. The metabolic profile of all the cell lines is significantly affected by both the individual and the combined treatments with MPA and BEZ.

The specific metabolic changes induced by the different drug treatments were further investigated by performing PCA on the ^1^H-NMR datasets of each cell line. The scores plots (PC1 vs PC2) obtained from these analyses (Figure 3) depict excellent grouping and separation for the different treatments in each of the three cell lines. It is noteworthy that, for all the cell lines, the treatments with and without BEZ are always separated along the first principal component.

For KG1a, the separation between different treatments along PC2 is well resolved and separates treatments with and without MPA (regardless of BEZ); however, the treatments containing MPA, and with or without BEZ, are on opposite sides of the PC2-axis null point. Separation in PC2 is good for K562 although not as clear as for KG1a. Furthermore, separation between BEZ treatment alone and combined MPA+BEZ treatment in HL-60 is incomplete, indicating less pronounced additional changes induced by combining the two drugs.

**Figure 4:**
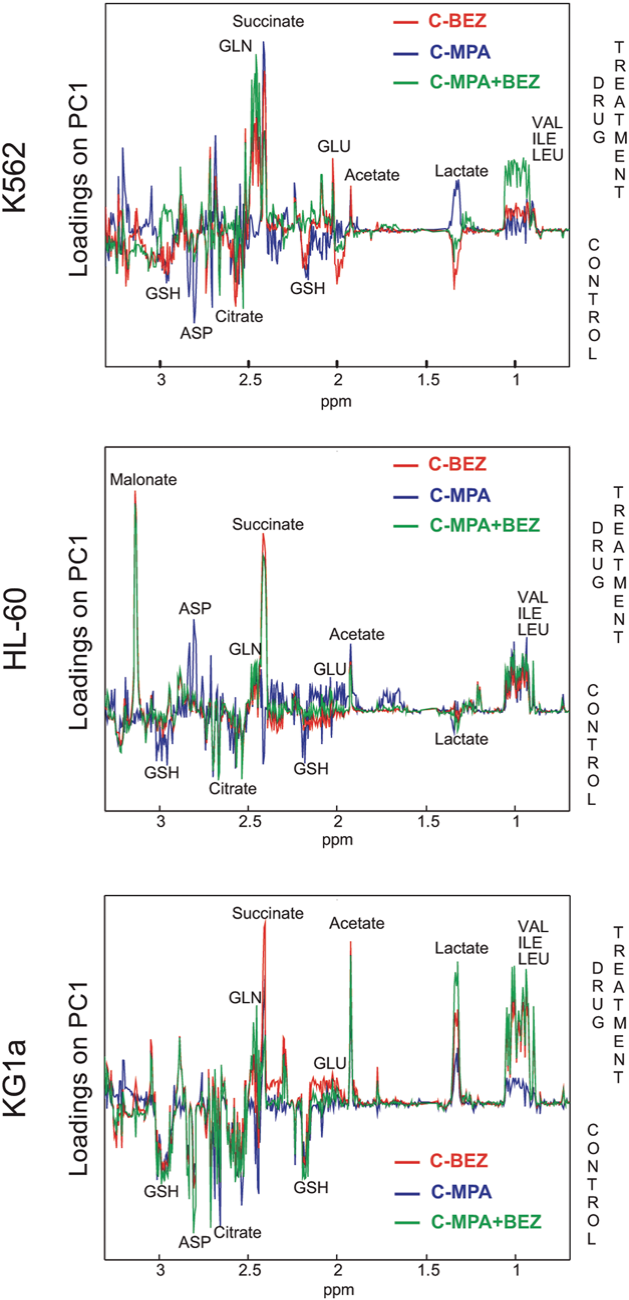
Section of the loadings plots (0.7–3.3 ppm) obtained from the pairwise PCA of solvent control versus MPA (blue line), BEZ (red line) and MPA+BEZ (green line) for K562, HL-60, and KG1a cells.

The excellent grouping and separation obtained from the aforementioned analysis encouraged us to further study the effects of the drug treatments on metabolic changes in the different cell lines and, eventually to try to identify the mechanism by which these drugs, and in particular the combination treatment, exert their function. To achieve this, each of the drug treatments were separately compared to the solvent control datasets (within each cell line) using PCA (scores plots obtained for KG1a cells are included in Figure S2(a–c)). In all cases this analysis led to a clear separation of the two treatments along PC1. These loadings were therefore used to identify discriminatory compounds for functional annotations (Figure 4).

The observed drug-induced changes depicted in Figure 4 indicated that a subset of metabolites was modulated in similar ways in all cell lines. Importantly, this was most marked when the combination treatment was compared to the controls suggesting that a common mechanism may underlie those changes. The concordant changes observed in response to combined treatment centered on metabolites intimately associated with the TCA cycle, some amino acids and other metabolites. For example, the amino acids valine, leucine and isoleucine were all present in higher concentrations in combination treatment samples as were glutamine, glutamate, glycine, succinate, and acetate. However, differential effects were also observed between non-differentiating KG1a and K562 cells when compared to HL-60 cells. For example fumarate accumulated in MPA+BEZ treated KG1a and K562 cells but was depleted in HL-60 cells. Perhaps in association with this aspartate accumulated in HL-60 cells but was depleted in KG1a and K562 cells. In addition, the response observed for glutamate is different for KG1a and K562 where MPA showed reduced levels and BEZ and combined treatment showed both increased levels whereas the effect of MPA was more dominant in HL-60 causing increased levels (in MPA and combined treatment) whereas BEZ alone caused lowered levels of glutamate.

### Addition of reactive oxygen to extract of non drug-treated cells results in drug like effects

**Figure 5:**
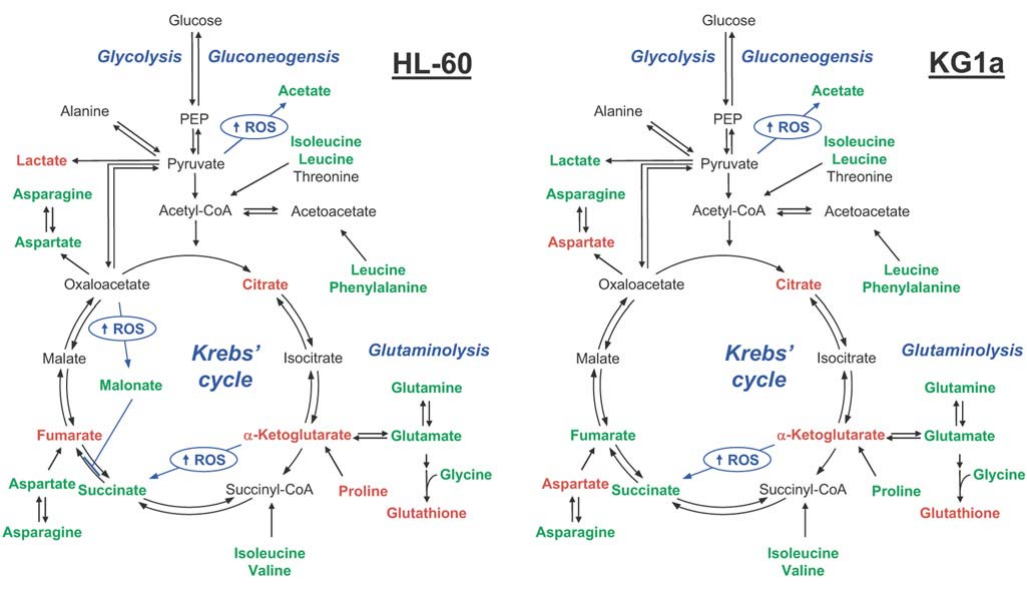
Schematic representation of the metabolic pathways showing the most relevant metabolic changes induced by MPA+BEZ drug treatment for the individual AML cell lines (HL-60 and KG1a). Metabolites in green/red have increased/decreased concentrations upon combined MPA+BEZ treatment.

**Figure 6:**
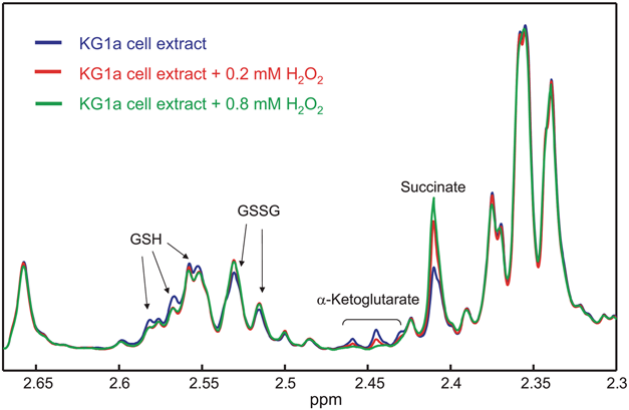
Section of ^1^H-NMR spectra (expanded between 2.3–2.67 ppm) of a de-proteinized extract of untreated KG1a cells (blue line) and after treatment with 0.2 mM (red line) and 0.8 mM (green line) H_2_O_2_.

Oxidative stress has been associated with TCA cycle dysfunction [22], [23]. In addition, previous reports have established that ROS mediate the non-enzymatic conversions including that of α-ketoglutarate into succinate [24]–[26]. As shown in Figure 5 and Figure S3, depletion of α-ketoglutarate and accumulation of succinate was a common drug response across all three cell lines. We therefore considered whether the perturbations of TCA intermediates seen in response to BEZ and MPA may relate to our previous observations of the ability of the drugs to generate ROS in AML cells [19]. To test this we monitored the *in vitro* effect of different concentrations (0.2, 0.8 and 2 mM) of hydrogen peroxide (H_2_O_2_) on KG1a cell extracts taken from non-drug treated cells. An expanded section of the acquired NMR spectra upon addition of H_2_O_2_ is shown in Figure 6 (for clarity the NMR spectrum obtained upon addition of 2 mM has been omitted because its profile was superimposed to 0.8 mM H_2_O_2_ NMR spectrum). Despite the apparent severity of this treatment the profile of the majority of metabolites remained unchanged. However, notable and highly selective changes where observed. As consistent with oxidative stress we observed depletion of reduced glutathione (GSH) and concurrent accumulation of oxidized glutathione (GSSG). Moreover, and consistent with the response to combined BEZ and MPA, H_2_O_2_ treatment was also associated with a clear reduction in α-ketoglutarate with concurrent gain of succinate.

In contrast, acetate and malonate don't accumulate in H_2_O_2_-treated extracts form KG1a cells owing to the transiently low concentration of pyruvate and oxaloacetate in cell extracts. However, *in vitro* treatment of oxaloacetate and pyruvate confirm that these conversions are in fact induced by hydrogen peroxide as shown in Figure S4.

## Discussion

### Relevance of metabolic profiling of cancer cells

Cancer cell lines are well established models to study specific cellular mechanisms characteristic for different types of cancer, usually by monitoring specific proteins and their actions. Recent advances in ‘omics’ technologies allow for more systematic investigations to characterize parameters like gene expression, protein and metabolite profiles [4], [20]. The advantage of ‘omics’ technologies lies in their multiparametric nature probing a large array of indicators at the same time. For the application of ‘omics’ technologies to the understanding of drug action, it is essential that effects of drugs produce a sensitive response which can be reproduced with sufficiently high quality. Here we have shown that metabolic profiling of cancer cells can not only identify phenotypic differences between cell lines but can also sensitively detect and distinguish changes in metabolite concentrations induced by relatively short exposure to differing drugs. It is important to mention that all treatments caused a distinct reproducible profile which is observed in unsupervised PCA. The schematics shown in Figure 5 and Figure S3 depict the commonalities and differences in changes in metabolites when each of the cell lines was treated with combined MPA+BEZ. These figures powerfully illustrate that a common metabolic response underlies the perception of the drugs in independent AML cell lines with markedly differing constitutive metabolomes.

For the functional relevance it is essential that two similar cell lines, K562 and KG1a, representing blockage at an early stage of maturation of the erythroid and myeloid lineages [21] showed a similar metabolic response to the MPA+BEZ treatment whereas HL-60 cells, which are closer to the mature granulocyte stage [21] showed a different overall response. HL-60 also showed a less pronounced combinatorial effect for combined MPA+BEZ treatment which was clearly distinguished from individual treatments in KG1a and K562.

### Functional annotation of the observed metabolic changes

Lowered α-ketoglutarate levels associated with increased succinate concentrations were consistently observed in all three cell lines when treated with combined BEZ and MPA, and this was recapitulated by the treatment of KG1a extracts with H_2_O_2_ (Figure 6). Thus it is most likely that ROS, generated as a consequence of drug treatment, are the direct cause of at least some of the metabolic changes we have observed.

ROS have also been demonstrated to convert pyruvate into acetate and oxaloacetate into malonate [25] and we have confirmed these conversions using *in vitro* tests (Figure S4). Although we were not able to detect pyruvate in our spectra, acetate was accumulated in extracts of all three cell lines when treated with combined BEZ and MPA. Owing to the low transient concentration of pyruvate acetate was not accumulated in H_2_O_2_-treated control extracts form KG1a cells. Taken together these observations suggest that flux through the pyruvate pool in HL-60, K562 and KG1a cells is rapidly maintaining a low steady-state concentration and that only in the presence of this sustained flux can the ROS-dependent conversion to a more stable acetate pool be detected.

Evidence of increased malonate was observed in extracts of HL-60 cells treated with either BEZ alone or combined BEZ and MPA but not in the other cell types. Again levels of oxaloacetate were below detection in all three cell lines. However, the ROS mediated conversion of oxaloacetate into malonate is confirmed by *in vitro* tests. Notably, the drug induced accumulation of malonate by HL-60 cells was associated with a concomitant fall in fumarate which again was not observed in either KG1a or K562 cells. Since malonate is known to inhibit succinate dehydrogenase it is likely that the malonate and fumarate changes seen exclusively in HL-60 cells are causally linked [24], [25]. This may also be linked to aspartate metabolism as fumarate concentrations were always inversely correlated to aspartate levels, possibly by an involvement of the urea cycle as supported by altered ornithine concentrations in HL-60. In fact, the treatment induced signature of succinate, malonate, fumarate, and aspartate represents a clear marker profile of apoptosing KG1a/K562 vs. non-apoptosing HL-60 cells.

The combined impact of dual BEZ and MPA treatment upon the TCA cycle are likely to impact upon broader aspects of mitochondrial function. This compromised mitochondrial functionality was also observed in the marked accumulation of metabolites which are essential for the biosynthesis of nucleotides, in particular glutamine and glycine, suggesting a hindered biosynthesis of purine and pyrimidine nucleotides, a known effect of mitochondrial dysfunction [27], [28].

As already highlighted the overall metabolic responses of KG1a and K562 cells were more similar to each other than to HL-60 cells. This reflects that the drugs primarily induce apoptosis in KG1a and K562 cells and differentiation in HL-60 cells. In HL-60 cells, purine and pyrimidine have been shown to have an important role for the progression of cellular differentiation [29]–[31]. However, it is not yet possible to determine whether the differential changes we have observed reflect early commitment to either apoptosis or differentiation, or indeed in some way ‘instruct’ these pathways. Future studies should address this important question.

In summary, the data presented in this study highlights the potential of ^1^H-NMR based profiling of drug response in cancer cells. Following other methods of functionally annotated profiling, the importance of metabolomics is that it describes the actual functional state of the cells. Metabolic changes observed in AML cell lines in response to BEZ/MPA treatment reflect the downstream effect of ROS on TCA cycle metabolites and on pyruvate and suggest that changes directly induced by ROS significantly contribute to the mechanism of action.

## Materials and Methods

### NMR sample preparation

KG1a, K562 and HL-60 AML cell lines were maintained in exponential proliferation in RPMI 1640 (Gibco-Invitrogen, Paisley, UK) medium with 10% fetal bovine serum (FBS, Gibco-Invitrogen) and 100 units/ml Penicillin and 100 µg/ml of Streptomycin (Gibco-Invitrogen).

5×10^7^ cells were treated with either solvent control (DMSO), 0.5 mM BEZ, 5 µM MPA or the combination of 0.5 mM BEZ and 5 µM MPA. Twelve replicate samples were prepared per treatment. After 24 hours, the cells were washed with PBS (Lonza Group Ltd., Basel, Swizzerland) and harvested by centrifugation. The pellets were immediately frozen in liquid nitrogen and stored at −80°C.

The extraction of intracellular metabolites from cell pellets was performed using a modified Bligh-Dyer procedure [32]. Samples were dried overnight in a centrifugal vacuum concentrator. The dried polar extracts were redissolved in 90% H_2_O/10% D_2_O (GOSS Scientific Instruments Ltd, Essex UK) prepared as 100 mM phosphate buffer (pH 7.0), containing 0.5 mM sodium 3-(trimethylsilyl)propionate-2,2,3,3-d4 (TMSP, Cambridge Isotope Laboratories) as internal reference.

### NMR experiments

A 500 MHz Bruker spectrometer equipped with a cryogenically cooled probe was used for 1D ^1^H and 2D ^1^H *J*-RES [33] NMR data acquisition. In both cases the water resonance was suppressed using excitation sculpting [34]. 1D spectra were acquired using a 90° pulse, and with 5 kHz spectral width, a relaxation delay of 3 s and 128 transients. 2D *J*-RES spectra were collected using a double spin echo sequence with 16 transients per increment and 32 increments. Strong coupling artifacts were suppressed by phase cycling [35]. Prior to Fourier transformation, 2D *J*-RES spectra were multiplied by a combined sine-bell/exponential window function in the direct dimension and by a sine bell function in the incremented dimension [36]. Skyline projections were calculated and spectra were aligned. Selected signals arising from residual solvents, TMSP and BEZ were excluded. Total spectral area was then normalised to unity and spectra were binned at 0.005 ppm (4 data points). The generalized-log transformation was applied prior to conducting the multivariate statistical analysis [37]. NMR data was processed using NMRLab [38] in the MATLAB (The MathWorks, Inc., Natick, MA) programming environment. PCA of the projected *J*-RES NMR spectroscopy data was carried out using PLS toolbox 4.1 (Version 4.1; Eigenvector Research, Manson, WA). NMR resonances of metabolites were assigned using the Chenomx NMR Suite (version 5.0; Chenomx Inc., Edmonton, Canada).

### Assessment of cell differentiation

Cell differentiation was assessed by measuring differentiation antigen expression after 24 and 96 hours treatment with combined MPA and BEZ using flow cytometry (Becton Dickinson FACS Calibur and Becton Dickinson Cell Quest software) and PE-CD11b conjugated antibodies for HL-60 and KG1a cells. FITC-Glycophorin-A was used to determine macrophage differentiation of K562 (Serotec).

### Assessment of Apoptosis by Annexin-V

Apoptosis was evaluated by measuring Annexin-V expression using the Annexin-V FITC kit (Becton Dickenson, Oxford, UK). Analyses were carried out by flow cytometry using a Becton Dickinson FACS Calibur utilising Cell Quest Pro software (Becton Dickenson, Oxford, UK).

### Jenner Giemsa staining of slides

Cytospins were prepared from 75-100 µl of culture. Slides were air-dried, methanol fixed and stained; First with Jenner staining solution (VWR) diluted 1/3 in 1 mM sodium phosphate buffer pH 5.6 (5 min) and second with Giemsa stain (VWR) diluted 1/20 in 1 mM sodium phosphate buffer pH 5.6 (10 min). Slides were dried and then mounted onto cover-slips using DePex (VWR, UK).

## Supporting Information

Figure S1 Loadings plots for the first principal component of the pairwise comparison for the 3 untreated cell lines. Blue line: loadings plot of K562 (positive loadings) versus KG1a (negative loadings); red line: loadings plot of K562 (positive loadings) versus HL60 (negative loadings). Metabolites showing the most intense changes are labeled on the plot (Lac, lactate; Ala, alanine; NAA, N-acetyl-aspartate; Cr, creatine; PCho, phosphocholine; MI, myo-inositol).
(0.37 MB EPS)

Figure S2 Scores plots for the pairwise analysis of solvent-control versus (a) MPA, (b) BEZ, and (c) MPA+BEZ treatment in the KG1a cell line; (d) section of the loadings plot obtained for the comparison of the solvent-control versus MPA (blue), BEZ (red), and MPA+BEZ(green) for KG1a.
(0.45 MB EPS)

Figure S3 Schematic representation of the metabolic pathways showing the most relevant metabolic changes induced by MPA+BEZ drug treatment for K562 AML cell line. Metabolites colored in green/red have increased/decreased concentrations in the combined MPA+BEZ treatment.
(0.34 MB EPS)

Figure S4 In vitro effect of H2O2 treated metabolites. Relative concentrations obtained from 1D 1H-NMR spectra of a mixture of oxaloacetate (a), alpha-ketoglutarate (b) and pyruvate (c) (4 mM) treated with different concentrations of H2O2. Oxaloacetate, alpha-ketoglutarate and pyruvate are quantitatively converted to malonate (d), succinate (e) and acetate (f), respectively.
(0.32 MB EPS)
