# Correction: Metabolomic Profiling of Drug Responses in Acute Myeloid Leukaemia Cell Lines

**DOI:** 10.1371/annotation/39584d38-04f5-4b37-bfd8-eae2318ec6f9

**Published:** 2009-04-01

**Authors:** Stefano Tiziani, Alessia Lodi, Farhat L. Khanim, Mark R. Viant, Christopher M. Bunce, Ulrich L. Günther

Some information was omitted from the funding statement. The correct statement should read: This work was funded by the Leukaemia Research Fund (LRF, UK), Marie Curie Transfer of Knowledge (ToK) award (MOTET), and the NERC Fellowship to MRV. (NER/J/S/2002/00618). The funders had no role in study design, data collection and analysis, decision to publish, or preparation of the manuscript. 

